# Mental health emergencies and COVID-19: the impact of ‘lockdown’ in the East Midlands of the UK

**DOI:** 10.1192/bjo.2021.973

**Published:** 2021-07-26

**Authors:** Harriet Elizabeth Moore, Aloysius Niroshan Siriwardena, Mark Gussy, Frank Tanser, Bartholomew Hill, Robert Spaight

**Affiliations:** School of Geography, Brayford Pool, DIRE Research Group, University of Lincoln, UK; School of Health and Social Care, University of Lincoln, UK; Lincoln Institute for Rural Health, University of Lincoln, UK; School of Engineering, Loughborough University, UK; East Midlands Ambulance Service NHS Trust, UK

**Keywords:** COVID-19, mental health, emergency medical data, social isolation, lockdown

## Abstract

**Background:**

The most immediate response of the research community to COVID-19 has been a focus on understanding the effects, treatment and prevention of infection. Of equal and ongoing importance is elucidating the impact of mitigation measures, such as lockdown, on the well-being of societies. Research about mental health and lockdown in the UK has predominately involved large surveys that are likely to encounter self-selection bias. Further, self-reporting does not constitute a clinical judgement.

**Aims:**

To (a) compare the age, gender and ethnicity of patients experiencing mental health emergencies prior compared with during lockdown, (b) determine whether the nature of mental health emergencies has changed during compared with before lockdown, (c) explore the utility of emergency medical service data for identifying vulnerability to mental health emergencies in real time during a pandemic.

**Method:**

A total of 32 401 clinical records of ambulance paramedics attending mental health emergencies in the East Midlands of the UK between 23 March and 31 July 2020 and the same period in 2019 were analysed using binary logistic regression.

**Results:**

People of younger age, male gender and South Asian and Black ethnicity are particularly vulnerable to acute mental health conditions during lockdown. Patients with acute cases of anxiety have increased during lockdown whereas suicide and intentional drug overdose have decreased.

**Conclusions:**

Self-reported data may underrepresent the true impact of lockdown on male mental health and ethnic minority groups. Emergency medical data can be used to identify vulnerable communities in the context of the extraordinary circumstances surrounding the current pandemic, as well as under more ordinary circumstances.

## Background

In the early weeks of March 2020, the World Health Organization addressed the mental health risks associated with the coronavirus disease 2019 (COVID-19) pandemic, encouraging people in isolation to maintain social networks virtually, practice mindfulness and limit access to news reports that might produce anxiety.^1^ The most immediate response of the research community to COVID-19 has, necessarily, been a focus on understanding the infection, its treatment and prevention.^2^ Understanding the impact of transmission mitigation strategies, such as social distancing and ‘lockdown’ restrictions, on the mental health and well-being of people and populations is of equal importance.^3^ Here we explore the demographic characteristics of mental health patients requiring ambulance attendance, and the changing nature of mental health emergencies during ‘lockdown’ in the East Midlands of the UK. Emergency medical service (EMS) data offer novel opportunities to understand the impact of the current pandemic on the health and well-being of populations in real time,^4^ as well as to optimise the delivery of strained healthcare services.

## Importance of the research

The COVID-19 pandemic is having adverse psychological effects on populations worldwide,^5,6^ including in the UK.^7^ Levels of anxiety and depression experienced by societies has increased over the course of the pandemic.^8^ Loneliness and isolation because of extended periods of lockdown mitigation strategies have been associated with declining mental health including increased depression, suicidal ideation and anxiety.^9,10^ However, measuring and quantifying the impact of a pandemic on community mental health is challenging. Empirical studies examining the psychological impact of social distancing during COVID-19,^11^ and other recent Severe Acute Respiratory Syndrome^12^ and Middle East Respiratory Syndrome^13^ pandemics, often rely on self-reported questionnaire data. This methodological approach is logistically convenient but problematic because the reliability of self-reported mental health data can be undermined by response bias. For example, the self-selecting nature of voluntary survey participation can result in non-response bias; women and healthier people tend to respond more readily than men and less healthy people,^14^ and men tend to underreport the severity of mental health conditions such as depression.^15^ Certainly, large-scale surveys about the impact of social distancing during COVID-19 on mental health conducted in China,^11,16^ the UK^7^ and elsewhere^17^ report that women have experienced greater psychological distress than men. Self-reported research also suggests that younger age may also be a risk factor for experiencing poor mental health during the pandemic.^18^ However, these results should be interpreted with caution as self-reporting, although an invaluable indicator of psychological symptoms, does not signify clinical assessment.

The methodological challenges of investigating the mental health impact of the current pandemic in real time reflect common limitations of research about social isolation and loneliness. Most research falls into two categories: examining the neurological effects of isolation on animals or examining the effect of perceived loneliness in human cohorts.^19^ Of studies about perceived loneliness, the overwhelming majority has relied on self-reported survey data.^20^ Further, studies rarely ask participants to record demographic information that would help elucidate vulnerabilities, such as ethnicity.^20^ In the UK, thus, clinical understandings of the mental health impact of loneliness and isolation in vulnerable communities are constrained by the subjective nature of data and lack of demographic complexity in respondents.

## Goals of this investigation

In addition to informing prehospital triage for mental health emergencies, EMS data, such as the ambulance data we utilise, offer three important opportunities to address gaps in reporting of mental health issues related to COVID-19,^21^ as well as understanding the effects of loneliness and isolation. First, it enables us to explore whether demographic characteristics including age, gender and ethnicity, of patients calling an ambulance with mental health emergencies have changed during lockdown compared with prior to lockdown. Second, ambulance data allow examination of change in mental health emergencies during lockdown compared with beforehand, offering insights into the evolving nature of mental health emergencies over the course of the pandemic. Understanding how these clinical conditions present during a pandemic can increase service providers’ awareness of the needs of vulnerable communities. Third, our data-set offers an opportunity to explore whether EMS data, which may be less affected by bias than self-reported data, are consistent with existing knowledge about groups such as ethnic minorities who are more vulnerable to mental health emergencies^22^ as they have been found to experience disproportionately higher rates of mental health illness compared with other ethnic groups.^23^

At the time of writing, the UK has experienced multiple extended periods of extraordinary restrictions, including prohibiting mixing between households and limiting physical social contact. We explore the impact of the first national lockdown in 2020 on community mental health by investigating whether demographic factors and the specific characteristics of mental health emergencies in this region are different during the first national lockdown compared with the same period during the previous year. We also consider the utility of EMS data for overcoming some of the limitations of purely self-reported mental health research. Managing the strain of community mental health on EMS systems involves optimising triage in the immediate future as well as reducing the prevalence of mental health emergencies over the longer term. EMS data may elucidate vulnerability, facilitating more tailored prehospital mental health services, as well as offering insight into the impact of social isolation on acute mental health emergencies more generally.

## Method

### Research aims, design and setting

The aims of the study were as follows.

- To explore the demographic characteristics of mental health emergencies during lockdown compared with before lockdown, including whether the age, gender and ethnicity of patients experiencing these emergencies has changed. The purpose was to identify vulnerable groups that may require additional assistance over extended periods of physical and social isolation.
- To investigate whether the nature of mental health emergencies has changed during compared with before lockdown.
- To explore the utility of EMS data for identifying vulnerability to mental health emergencies in real time during a pandemic as an alternative to relying on self-reporting that is affected by self-selection bias.

National lockdown to mitigate the transmission of COVID-19 in the UK was first introduced on 23 March 2020 and was in place until 4 July. Restrictions were progressively eased over the month of July. A three-tier system designating specific restrictions for individual regions was introduced in October in response to rapidly rising rates of transmission.^24^ We used a before and after observational study design analysing routine ambulance clinical data from the East Midlands of the UK between 23 March and 31 July 2020 and the same period in 2019 to evaluate the impact of lockdown on community mental health in the East Midlands.

**Fig. 1:**
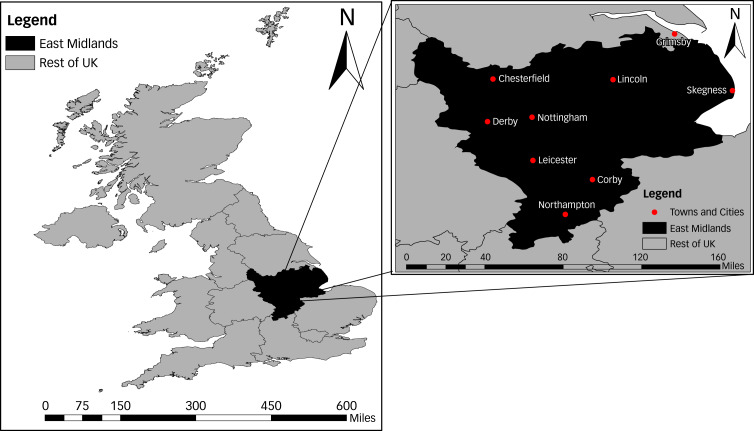
Map of the UK highlighting the East Midlands region, including the locations of prominent towns and cities.

The East Midlands, located in the Central Eastern part of England, spans an area of 15 627 km^2^ (Fig. 1). The estimated total population of the region is 4.8 million and include the populous urban areas of Derby, Leicester, Lincoln, Northampton and Nottingham.^25^ The proportion of the population identifying as other than ‘White UK’ in the East Midlands is low (14.6%) compared with the national average (20.2%).^26^ In 2016, 18.5% of people in the region lived in the most deprived quintile.^27^ This region is also the third most rural region in England.^28^

The routine clinical data collected by the East Midlands Ambulance Service NHS Trust (EMAS), comprise clinical impressions of medically trained ambulance paramedics attending emergency calls, including those related to mental health. Mental health emergencies involve severe mental health presentations. For each individual mental health emergency medical professionals have recorded clinical impressions including anxiety, depression, psychosis, acute behavioural disturbance, intentional drug overdose and attempted suicide.

### Population

The research involves the population of the East Midlands of the UK including all patients who have been attended by ambulances for mental health-related emergencies following 999 calls to ambulance services between 23 March and 31 July of 2019 and 2020.

### Measures

The Appendix summarises the measures included in the research. Although the study design is quasi-experimental, data collection was retrospective and observational. The data-set obtained from EMAS includes patient records collated routinely by paramedics attending emergencies following 999 calls, such as the age, gender and ethnicity of patients. The ethnicity categories described in this study reflect key distinctions outlined in the UK Government ethnicity classification.^29^ In some cases, paramedics were unable to record ethnicity, such as when making an enquiry was inappropriate given the condition of the patient.

Clinical categories were determined based on the ‘primary impression’ of trained paramedic clinicians attending patients. Primary impressions include the professional judgement of a trained paramedic clinician as well as the mental health history reported by the patient or other individuals who are known to the patient and are present at the time of ambulance attendance, such as carers and family members. Thus, the preliminary impression of the paramedic clinician reflects the presenting symptoms of the patient at the time of the emergency as well as medical history. Therefore, ambulance records capture severe mental health symptoms as well as underlying conditions culminating in medical emergencies.

### Data handling and cleaning

The data-set obtained by EMAS, comprised 32 401 individual patient records of mental health emergencies-related 999 calls (this research was approved by the NHS Health Research Authority, IRAS ID: 264573). Records were a subset of routinely collected data including the date 999 calls were received by EMAS, gender, age, ethnicity and a clinical impression of the nature of the mental health emergency.

### Data analysis

A binary logistic regression was conducted to explore differences in the demographic characteristics of patients and the nature of mental health emergencies during the lockdown compared with before lockdown. To control for the effects of seasonality^30^ we compared records collated during the first national lockdown between 23 March to 31 July 2020 to records for the same time period in 2019. It should be noted that time series analysis is often used to explore trends occurring over smaller temporal graduations. We compare trends between two ‘conditions’; before lockdown and during lockdown. Logistic regression was selected for this purpose. The regression output was also used to consider whether EMS data reflected wider understanding about the relationship between demographic factors and mental health vulnerability.

## Results

The statistical analysis reported in the following addresses: the first aim of the research related to whether the demographic characteristics of patients experiencing mental health emergencies were different during the first phase of national lockdown in the UK compared with the same period for the year prior, and the second aim of the research about whether the nature of mental health emergencies occurring during lockdown were different compared with the year prior. The third aim about investigating the utility of EMS data in real time during an extended period of forced isolation, like a pandemic, is considered in the discussion.

### Descriptive statistics

The proportion of incidents occurring in 2019 compared with 2020 by gender, ethnicity, and the clinical impressions of paramedics are reported in Table 1. Of 32 401 records of mental health emergencies, 1629 contained missing information. Therefore, 30 772 were included in the final regression model. On average, the age of patients experiencing mental health emergencies between 23 March and 31 July 2019 (mean 42.39, s.d. = 20.18) was younger than the age of patients for the same period in 2020 (mean 44, s.d. = 20.49).

**Table 1:** The number and proportion (%) of mental health emergencies occurring between 23 March and 31 July in the years 2019 and 2020 by gender, ethnicity and clinical impressions. Total *N* for factors varies due to missing data. Proportions (%) are rounded to nearest full number; as a result the total proportion for clinical impressions exceeds 100%.

Factor	2019		2020	
	*n*	%	*n*	%
Gender				
Female	8540	58	9979	57
Male	6225	42	7481	43
Transgender	57	<1	51	<1
Ethnicity				
White British	12 789	91	14 982	89
White other	422	3	583	4
South Asian	454	3	649	4
Other Asian	10	<1	11	<1
Black	128	1	216	1
Arabic	<10	<1	13	<1
Mixed	256	2	308	2
Other	<10	0	<10	<1
Clinical impressions				
Other mental health problem	2167	15	2338	13
Acute behavioural disturbance	222	2	260	2
Anxiety	5219	36	7746	44
Attempted suicide	1339	9	1232	7
Depression	2318	16	2541	15
Intentional drug overdose	3227	22	3079	18
Sectioning under Mental Health Act	287	2	354	2

### Regression analysis

A binary logistic regression analysis was conducted to investigate the impact of the first phase of national lockdown on mental health emergencies. The analysis examined how well the demographic and clinical characteristics of patients predicted mental health emergencies during the first national lockdown between 23 March to 31 July 2020 compared with the same time period of the previous year.

The results indicated that age, two categories of ethnicity, one category of gender, and three categories of clinical impressions were significant predictors of mental health emergencies during the first national lockdown compared with the same period in 2019, (χ^2^ = 273.77, d.f. = 16, *P* = <0.001). The model correctly predicted 26.2% of emergencies occurring in 2019 and 78.9% of emergencies occurring during national lockdown in 2020, giving an overall prediction accuracy of 54.8%.

Table 2 displays the binary logistic regression results for all independent variables included in the model. The reference categories for categorical variables included in the model were as follows: ‘female’ for gender, ‘White British’ for ethnicity, and ‘other mental health problem’ for clinical impressions. Compared with instances of mental health emergencies occurring in 2019, those occurring during the first national lockdown were more likely to be associated with older age, male gender and South Asian and Black ethnicity. Mental health emergencies occurring during lockdown were more likely to be associated with anxiety, which was the strongest predictor, and less likely to involve attempted suicide and intentional drug overdose.

**Table 2:** Binary logistic regression for predicting mental health emergencies occurring during lockdown compared with the same period in the year prior to lockdown (2019)

	*B*	s.e.	Wald	d.f.	Exp(*B*)	95% CI
Gender						
Age	**0**.**001**	**0**.**001**	**5**.**53**	**1**	**1**.001**	**1.00–1.00**
Male	**0**.**05**	**0**.**02**	**4**.**8**	**1**	**1**.05**	**1.00–1.10**
Transgender	−0.17	0.19	0.71	1	0.85	0.58–1.25
White other	0.12	0.07	3.28	1	1.13	0.99–1.28
South Asian	**0**.**14**	**0**.**06**	**5**.**23**	**1**	**1**.16**	**1.02–1.31**
Ethnicity						
Other Asian	−0.11	0.44	0.06	1	0.9	0.38–2.12
Black	**0**.**34**	**0**.**11**	**8**.**91**	**1**	**1**.4*	**1.12–1.75**
Arabic	0.22	0.45	0.23	1	1.24	0.51–2.90
Mixed	0.04	0.09	0.24	1	1.04	0.88–1.24
Other	21	0.23044	0.00	1	1	0.00–0.00
Clinical impressions						
Acute behaviour disturbance	0.1	0.1	0.95	1	1.1	0.90–1.34
Anxiety	**0**.**28**	**0**.**04**	**56**.**76**	**1**	**1**.32*	**1.23–1.42**
Attempted suicide	−**0**.**15**	**0**.**05**	**8**.**98**	**1**	**0**.86*	**0.78–0.95**
Depression	0.02	0.04	0.15	1	1.02	0.94–1.11
Intentional drug overdose	−**0**.**12**	**0**.**04**	**6**.**82**	**1**	**0**.9*	**0.83–0.97**
Sectioning under the Mental Health Act	0.12	0.09	1.97	1	1.13	0.95–1.34

Predictor variables include age, gender, ethnicity and clinical impressions. Statistically significant predictor variables are indicated in bold.

* Statistically significant at *P* < 0.01.

** Statistically significant at *P* < 0.05.

## Discussion

The global state of emergency precipitated by COVID-19 has exposed the vulnerable subgroups of the population to multifaceted threats to well-being and increased the strain on EMS systems to provide specialised care for patients experiencing acute mental health emergencies. Others^31^ have noted the dual threat of the biological impact of the virus on the one hand, and on the other hand the exacerbation of non-communicable diseases related to physical inactivity during lockdown, such as cardiovascular conditions. We suggest that the mental health crisis adds a third dimension to the complex health risks associated with COVID-19.

We used ambulance data to explore vulnerability to mental health emergencies in the context of a pandemic during an extended period of lockdown that has been widely associated with increasing loneliness and social isolation.^9,10^ In the remainder of this paper we identify vulnerable groups, consider how well ambulance data reflects known patterns of vulnerability, highlight shifts in the nature of mental health emergencies during lockdown, and offer recommendations for future research and the operation of EMS systems.

### Identifying vulnerability: do emergency calls reflect known patterns?

Our results suggest that younger age and male gender predict mental health emergencies during the first national lockdown compared with the prior year. In contrast, research based on self-reported measures of mental health suggests that younger age^18^ and female gender^7,17^ are associated with greater psychological distress during the current pandemic. This is consistent with diagnostic statistics; women are more likely than men to be diagnosed with a common mental health condition, particularly anxiety. However, men tend to underreport psychological distress, and are less likely to help-seek, resulting in the underrepresentation of men in diagnostic statistics.^32^ Men^7^ and people of older age^33^ are also less likely to participate in voluntary mental health surveys. In the UK, men account for three-quarters of all reported suicides,^22^ suggesting that service use, diagnosis and help-seeking data are not an accurate representation of male mental health. Ambulance data indicates severe symptoms and acute psychological distress. Compared with more moderate or manageable experiences of distress, mental health emergencies reflect the culmination of multiple stressors.^34^ It is possible that social isolation related to lockdown escalates distress that is ordinarily underreported. Thus, ambulance data may be a more objective reflection of male mental health compared with self-reporting.

Our results also suggest that social distancing and lockdown may exacerbate pre-existing inequalities related to ethnicity. Ethnic minorities in the UK tend to experience higher rates of mental ill health compared with dominant ethnicities, such as White British.^22^ Compared with White British ethnicity, South Asian and Black ethnicity were found to predict mental health emergencies occurring during the first national lockdown. These trends reflect known associations between ethnicity and mental health vulnerability, suggesting that ambulance data may be a more objective measure of the impact on mental health compared with self-reported voluntary surveys. A social survey of 53 351 UK residents was conducted in April of this year including the 12-item General Health Questionnaire to measure mental health.^7^ No association between ethnicity and mental health was found. However, the response rate of people from ethnic minority groups in the UK to voluntary surveys such as NHS patient surveys is typically low compared with other ethnicities.^35^

Taken together, our analysis suggests that ambulance data does reflect well-understood patterns of mental health vulnerability related to ethnicity, and may be a more objective measure of vulnerability related to age and gender compared with self-reported data that is typically susceptible to self-selection bias.^36^

### The nature of mental health emergencies during lockdown

Our data indicate that during lockdown the proportion of mental health emergencies for attempted suicides and intentional drug overdose decreased compared with the same period in the year preceding the pandemic. These observations are consistent with those of our EDGE Consortium colleagues in Ontario, Canada, who found that rates of suicide^37^ and drug overdose (Graham, L. Rhiannon, C., Melissa, P., Rick, F., Brent, M., Robert, S., A. Niroshan, S., and Gina A., personal communication, 16th April, 2021) decreased during lockdown. In contrast, a recent study involving similar ambulance data and methodological approach in the USA concluded that suicide and overdose rates have significantly increased since the beginning of the pandemic.^38^ Importantly, the study examined rates over a comparable period to our research during the early months of the pandemic. Over this period in the UK a full national lockdown was imposed. By comparison, over the same period in the USA, lockdown was introduced on a state-by-state basis, with some states entering lockdown as late as April 2020. It is possible that varying rates between the UK and the USA reflect access and opportunity differences. Reduced rates of suicide and intentional drug overdose in the UK may be related to the introduction of lockdown and reduced access to harmful substances, as well as reduced access to prescription medication that is often associated with intentional overdose.^39^

These trends in no way diminish the impact of social isolation on community mental health. Rather, our observations may indicate the need to support vulnerable communities to a greater degree as lockdown restrictions are eased, particularly in the context of rising unemployment and financial constraint. The wider psychological impact of the pandemic on community mental health may prove to be a sleeping dragon in the wake of easing restrictions.

In contrast to attempted suicide and intentional drug ovderdose-related emergencies, the proportion of emergencies for anxiety increased during lockdown compared with the same period in 2019. Others have observed that the prevalence of self-reported anxiety has risen over the duration of the pandemic.^7^ Our findings suggest that mental health emergencies reflect the self-reported experience of the wider UK population; anxiety has increased during lockdown.^7^

### Limitations

Factors other than age, gender and ethnicity are likely to influence the presentation of severe mental health symptoms in a population. In the UK common mental health disorders are more prevalent in deprived compared with more affluent, and urban compared with more rural, communities.^40^ The impact of physical and social isolation is also likely to be compounded by financial instability associated with furlough and redundancies.^41^ Thus, further research should explore the relationship between the demographic, socioeconomic and geographic characteristics of populations and vulnerability to mental health emergencies.

Ambulance data represent a subset of mental health emergencies occurring within a population; people living within proximity to a hospital or other medical facility may access services directly without calling an ambulance. Further, help-seeking behaviour varies demographically and in relation to mental health literacy. Thus, ambulance attendance represents a proportion of mental health emergencies occurring during lockdown rather than the true frequency. Further, while EMAS records include the clinical assessments of trained paramedics, these assessments are not diagnostic. Clinical impressions include the self-reported experience of patients as well as more objective assessments of mental state made by ambulance clinicians. However, linkage with hospital or primary care data would be required to determine whether these patients who were attended to by ambulance clinicians have current diagnoses of mental health conditions.

### Implications for practice and research

Overall, our findings suggest some avenues for clinical practice as well as EMS systems in the study region, inducing specialised paramedic training for mental health conditions that are likely to increase in frequency and severity during a national emergency, and possibly the need to tailor service provision for people of ethnic minority background. However, the East Midlands represents a microcosm of the UK in terms of ethnic diversity, urban–rural dynamics and deprivation. Thus, we highlight some implications for future research, mental health practitioners and EMS systems in the UK more generally related to both responding to mental health emergencies and reducing the frequency of emergencies occurring.

- Clinical practitioners may wish to consider the shifting needs of vulnerable patients as lockdown restrictions are eased and opportunities to engage in harm behaviours are restored, particularly with regards to accessing illicit harmful substances and the potential misuse of prescribed mental health medications.
- Demographic characteristics including younger age, male gender, South Asian and Black ethnicity were associated with mental health emergencies during the first national lockdown. In-depth qualitative research is needed to understand why, including the intersection of other risk factors such as unemployment, prior health conditions and the role of social support networks.
(c) Greater focus is needed on the impact of isolation on male mental health, and consideration of ways to support young men beyond traditional social service models that rely on active help-seeking.
- EMS data may be a more objective measure of how loneliness and social isolation have an impact on mental health compared with self-reported survey data. Researchers should consider drawing on these data-sets to supplement voluntary social surveys to ensure all demographic groups are accurately represented.
- Data linkage between EMS data-sets, general practitioner records and hospital records could determine the proportion of mental health emergencies occurring in groups with pre-existing diagnoses compared with the proportion of new, emerging psychiatric conditions.
- Spatial analysis of emergency mental health data could identify social, environmental and economic factors related to household vulnerability using proxy indicators, such as the Index of Multiple Deprivation (details available from the authors on request).
- Considering the cost and time associated with data linkage, analysing ambulance data provides immediate, real-time insight into vulnerable demographics. National coordination and compilation of ambulance data, and other emergency medical data, could facilitate a more accurate appraisal of mental health emergencies, and identification of vulnerable groups during a pandemic, as well as in ordinary circumstances.

Ambulance data show how mental health emergencies have unfolded in the UK over the course of the pandemic. However, the implications of our research extend beyond the immediate crisis. The last national UK Community Life Survey found that 1 in 20 adults over the age of 16 reported feeling lonely ‘often or always’.^42^ In some cases, feelings of loneliness and isolation will precipitate anxiety, depression, disturbed behaviour, and in extreme cases self-harm or suicide. We demonstrate that novel emergency medical data can be used to identify vulnerable populations. We encourage greater attention to the methods we have used in this study. With reference to the first extended period of lockdown in the UK Pierce and colleagues^7^ suggested,

‘*As the economic consequences of lockdown develop, when furloughs turn to redundancies, mortgage holidays expire, and recession takes effect… it is reasonable to expect not only sustained distress and clinically significant deterioration in mental health for some people, but emergence of well described long-term effects of economic recession on mental health including increasing suicide rates and hospital admissions for mental illness.*’

We support the appeal of others^21^ for funding agencies to increase opportunities for the research community to focus greater attention on the third face of this pandemic; the mental health emergency, including the efforts of health systems to keep pace with the psychiatric needs of communities. The mental health impact of the pandemic, and associated mitigation methods are likely to endure well beyond the cessation of lockdown conditions.^7^ EMS data provide an evidence base for informing prehospital and hospital emergency services, as well as identifying avenues for prevention. It is our hope that the insights and recommendations detailed here inform investment in essential research and mental health services to support vulnerable communities during these turbulent times.

## Data Availability

The data that support the findings of this study are not publicly available because of restrictions. The data contain spatial information that could compromise the privacy of research participants and was obtained through a formal agreement with the East Midlands Ambulance Service.

## Appendix

### Research measures obtained from East Midlands Ambulance Service NHS Trust (EMAS) data-set, including date of 999 call received, age, gender, ethnicity and clinical impressions of the mental health emergency

**Table:**

Measure	Data
Age	Years
Gender	Categories: female, male, transgender
Ethnicity	Categories: White UK^a^, other White^b^, south Asian, other Asian, Black, Middle Eastern, mixed, other
Clinical impression	Categories: acute behavioural disturbance, anxiety, attempted suicide, depression, intentional drug overdose, psychosis, sectioned under the Mental Health Act, other mental health problem^c^

a. For some patients, multiple ethnicities were recorded for a single patient, such as ‘White British/Indian’. In consultation with the EMAS Head of Clinical Research and Audit we were advised to collapse each record to a single ethnicity category to be consistent with the approach followed by EMAS. Thus, in the example above, the patient record was coded as ‘White UK’.

b. ‘Other White’ refers to all non-UK White individuals, predominately individuals from Eastern Europe.

c. ‘Other mental health problem’ refers to patients where the nature of the mental health emergency has not been specified by the attending paramedic and may include instances where the primary problem was unable to be diagnosed on site.
